# ATPase-regulated autophagosome biogenesis

**DOI:** 10.1080/15548627.2023.2255967

**Published:** 2023-12-27

**Authors:** Viola Nähse, Kay O. Schink, Harald Stenmark

**Affiliations:** a Centre for Cancer Cell Reprogramming, Faculty of Medicine, University of Oslo, montebello, Oslo Norway; b Department of Molecular Cell Biology, Institute for Cancer Research, Oslo University Hospital, Montebello, Oslo, Norway; c Department of Anatomy and Stem Cells and Metabolism Research Program, Faculty of Medicine, University of Helsinki, Helsinki, Finland; d Department of Molecular Medicine, Institute of Basic MedicalSciences, University of Oslo, Oslo, Norway

**Keywords:** Aggrephagy, Atpase, autophagosome, micronucleophagy, mitophagy, omegasome

## Abstract

Omega-shaped domains of the endoplasmic reticulum, known as omegasomes, have been suggested to contribute to autophagosome biogenesis, although their exact function is not known. Omegasomes are characterized by the presence of the double FYVE domain containing protein ZFYVE1/DFCP1, but it has remained a paradox that depletion of ZFYVE1 does not prevent bulk macroautophagy/autophagy. We recently showed that ZFYVE1 contains an N-terminal ATPase domain which dimerizes upon ATP binding. Mutations in the ATPase domain that inhibit ATP binding or hydrolysis do not prevent omegasome expansion and maturation. However, omegasome constriction is inhibited by these mutations, which results in an increased lifetime and thereby higher number of omegasomes. Interestingly, whereas *ZFYVE1* knockout or mutations do not significantly affect bulk autophagy, selective autophagy of mitochondria, protein aggregates and micronuclei is inhibited. We propose that ATP binding and hydrolysis control the di- or multimerization state of ZFYVE1 which could provide the mechanochemical energy to drive large omegasome constriction and autophagosome completion.

Even though the core components of the autophagy machinery, the ATG proteins, have been identified and characterized, the biogenesis of the autophagosome remains incompletely understood. The recent discoveries that ATG2 is a lipid channel and ATG9 is a lipid scramblase have reinforced the idea that lipid transfer from the endoplasmic reticulum (ER) contributes importantly to the elongation and expansion of the phagophore, which eventually closes to form an autophagosome, but how does the phagophore acquire its characteristic cup shape prior to closure? An interesting hypothesis is that ER domains known as omegasomes, characterized by the protein ZFYVE1, help in shaping the nascent phagophore. However, the exact involvement of omegasomes in autophagosome biogenesis has remained elusive, and experiments with depletion of ZFYVE1 have failed to yield any autophagic phenotype. In a recent paper we have presented data that shed new light on the function of omegasomes and ZFYVE1 in autophagosome biogenesis [1].

ZFYVE1 is characterized by the presence in its C terminus of two non-canonical FYVE domains with weak but measurable affinity for phosphatidylinositol-3-phosphate/PtdIns3P. The N terminus of ZFYVE1 had not been characterized until recently, but we now find that this part of ZFYVE1 contains an ATPase domain. Interestingly, the ATPase domain forms a dimer in the presence of a non-hydrolyzable ATP analog but not with ADP. Curiously, a ZFYVE1 mutant with strongly reduced ATPase activity fails to form dimers, which suggests that dimerization might be required for ATPase activity. A structural characterization of ZFYVE1 dimers is required in order to fully understand the relationship between dimerization and ATP hydrolysis.

Live microscopy of cells expressing fluorescently tagged ZFYVE1 proteins together with autophagic markers show that ZFYVE1 can initially be detected as a small dot which expands to form the characteristic omega shape, with the cargo receptor SQSTM1 and the phagophore markers MAP1LC3B and WIPI2 inside. When the ZFYVE1-marked omegasome reaches its maximal size, the phagophore starts to extrude, and the omegasome gradually constricts to form one or two ZFYVE1 dots. Importantly, whereas ZFYVE1 mutants defective in ATP binding or hydrolysis form omegasomes with similar kinetics as wild-type ZFYVE1, these omegasomes show strong delays in their constriction. This delay results in longer omegasome lifetimes and consequently a higher number of omegasomes.

How might delayed omegasome constriction affect autophagosome biogenesis? If one assumes that omegasome constriction promotes phagophore constriction, autophagosome closure and thereby autophagic flux would depend on omegasome constriction. An argument against this hypothesis is the finding that starvation-induced MAP1LC3B lipidation, an indicator of bulk autophagic flux, is not affected by ZFYVE1 depletion. In accordance with this, we cannot detect any significant decrease in starvation-induced bulk autophagy upon ZFYVE1 depletion or mutagenesis. By contrast, however, the levels of SQSTM1 increase under these conditions, and autophagic degradation of mitochondria and puromycin-induced protein aggregates is inhibited. In addition, knockout of *ZFYVE1* leads to increased numbers of micronuclei, suggesting that autophagy of micronuclei is inhibited. These results indicate that ZFYVE1, contrary to expectations, is important for selective autophagy.

Considering that omegasomes are typically induced by amino acid starvation, how can we explain that ZFYVE1 is required for selective and not for bulk autophagy? ZFYVE1 might be a passive component of omegasomes in general, but its activity becomes important for their constriction only when their size is sufficiently large, as is typically the case with selective autophagy. This suggests the presence of additional factors that accomplish constriction of smaller omegasomes, and it will be interesting to identify these.

A remaining question is how the ATPase activity of ZFYVE1 can control omegasome constriction. ZFYVE1 bears structural similarity to the large GTPase DNM (dynamin), which is thought to mediate completion of endocytic vesicles via formation and constriction of helical assemblies at membrane necks. Constriction of these polymeric assemblies is achieved by repeated cycles of nucleotide loading and hydrolysis. Like DNM, ZFYVE1 contains membrane binding domains, and similar nucleotide cycles of ZFYVE1 might drive omegasome constriction.

With the new insight into ZFYVE1 function and the recently emerging concept of ATG9-containing vesicles as seeds for phagophores, it is possible to suggest a model for phagophore biogenesis (Figure 1). When an ATG9-containing vesicle contacts the ER, ATG2-mediated lipid transfer causes the vesicle to grow, and the scramblase activity of ATG9 ensures equilibration across the bilayer. ZFYVE1 is recruited at this stage, and together with yet unidentified ER proteins it forms an omegasome that sculpts the forming phagophore resulting from flattening of the ATG9 vesicle. Ultimately, if the omegasome is sufficiently large, the ATPase activity of ZFYVE1 is essential for constricting the omegasome, thereby causing release of the phagophore. Eventual phagophore closure is completed by ESCRT proteins.

**Figure 1.**
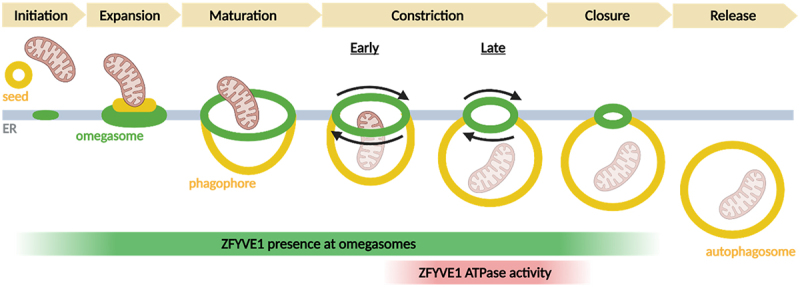
Stages of autophagosome biogenesis at omegasomes. ZFYVE1/DFCP1 is present at omegasomes from the initiation stage, but its ATPase activity is required for late constriction. Created with Biorender.com.
